# Serum and Salivary IgE, IgA, and IgG_4_ Antibodies to *Dermatophagoides pteronyssinus* and Its Major Allergens, Der p1 and Der p2, in Allergic and Nonallergic Children

**DOI:** 10.1155/2011/302739

**Published:** 2011-10-05

**Authors:** Diego O. Miranda, Deise A. O. Silva, Jorge F. C. Fernandes, Meimei G. J. Queirós, Hamilton F. Chiba, Leandro H. Ynoue, Rafael O. Resende, Janethe D. O. Pena, Sun-Sang J. Sung, Gesmar R. S. Segundo, Ernesto A. Taketomi

**Affiliations:** ^1^Laboratory of Allergy and Clinical Immunology, Institute of Biomedical Sciences, Federal University of Uberlândia, Avenue Pará 1720, Campus Umuarama, 38400-902 Uberlândia, MG, Brazil; ^2^Laboratory of Clinical Analysis, 280 Padre Felix Street, 75523-220 Itumbiara, GO, Brazil; ^3^Laboratory of Molecular Biology, Institute of Biomedical Sciences, Federal University of Uberlândia, Avenue Pará 1720, Campus Umuarama, 38400-902 Uberlândia, MG, Brazil; ^4^Center for Immunity, Inflammation and Regenerative Medicine, Department of Internal Medicine, Health Sciences Center, School of Medicine, University of Virginia, 22908-0395 Charlottesville, VA, USA

## Abstract

Allergic rhinitis (AR) is a public health problem with high prevalence worldwide. We evaluated levels of specific IgE, IgA, and IgG4 antibodies to the *Dermatophagoides pteronyssinus* (Dpt) house dust mite and to its major allergens (Der p1 and Der p2) in serum and saliva samples from allergic and nonallergic children. A total of 86 children were analyzed, from which 72 had AR and 14 were nonallergic healthy children. Serum IgE and serum/salivary IgG4 levels to Dpt, Der p1, and Der p2 were higher in allergic children whereas serum/salivary IgA levels to all allergens were higher in nonallergic children. IgE levels positively correlated with IgG4 and IgA to all allergens in allergic children, while IgA levels negatively correlated with IgG4 to Dpt and Der p1 in nonallergic children. In conclusion, mite-specific IgA antibodies predominate in the serum and saliva of nonallergic children whereas mite-specific IgE and IgG4 are prevalent in allergic children. The presence of specific IgA appears to have a key role for the healthy immune response to mucosal allergens. Also, specific IgA measurements in serum and/or saliva may be useful for monitoring activation of tolerance-inducing mechanisms during allergen specific immunotherapeutic procedures, especially sublingual immunotherapy.

## 1. Introduction

Allergic rhinitis (AR) is a global public health problem and it is gaining importance due to the rapid increase in its prevalence worldwide [1]. In Brazil, in a study using the International Study of Asthma and Allergies in Childhood (ISAAC), Solé et al. [2] found an average prevalence of rhinitis from 25.7% in groups of children aged 6-7 years and 31.7% between adolescents aged 13-14. According to ARIA (allergic rhinitis and its impact on asthma), AR is clinically defined as an inflammation of the nasal mucosa, mediated by IgE after exposure to allergens, and the symptoms occur most frequently for two or more days for more than 1 hour [1, 3]. Recently, other allergen-specific antibodies such as IgG, IgG4, and IgA have been reported to be involved during the course of allergic diseases [4]. 

House dust mites (HDMs), especially *Dermatophagoides pteronyssinus*, are considered an important source for allergen sensitization and they are a major risk factor for allergic respiratory diseases in genetically predisposed patients [5]. Their allergens are divided into groups according to their biochemical composition, homology, and molecular weight. Nowadays, 20 allergens have been described for *D. pteronyssinus* [6]. The group 1 (Der p1, 25 kDa) allergens are located in high concentration in faecal pellets [7], while those of the group 2 (Der p2, 14 kDa) are most found in components of the mite body [5]. Der p1 and Der p2 are considered major allergens of *D. pteronyssinus* due to being recognized by more than 80% of mite-sensitive patients [8]. 

Allergens derived from *D. pteronyssinus* are potential immunogens that are recognized by IgE, IgA, and IgG subclasses in allergic individuals [4]. In most allergic children, the levels of serum IgA are reduced and do not increase with age, as occurs in nonallergic infants. Nonatopic individuals respond with normal production of IgA against exposure to environmental allergens, which would trigger the mechanism of immune exclusion, preventing binding of allergens to IgE-producing cells. In this context, in children with IgA deficiency, this mechanism of immune exclusion would not occur, allowing the contact of antigen with IgE-producing plasma cells and thus triggering the mechanisms of type I hypersensitivity [9, 10]. While the elevation of serum IgE levels in response to environmental allergens is a distinctive feature of atopy, allergen-specific IgG antibodies to these allergens are detected in serum and saliva in both atopic and nonatopic individuals [11]. IgG1 and IgG4 are the main subclasses of allergen-specific IgG and the predominance of a certain class depends on the degree of exposure to allergen [11]. 

The present study aimed to evaluate the levels of IgE, IgA, and IgG4 antibodies specific to *D. pteronyssinus* and to its major allergens, Der p1 and Der p2, in samples of blood serum and saliva from allergic and nonallergic children.

## 2. Methods

### 2.1. Subjects

A total of 72 children aged 5 to 15 years, male and female, with perennial allergic rhinitis with or without intermittent or persistent, mild-to-moderate asthma were recruited from the Program of Asthma and Rhinitis Control of the Public Health System of Itumbiara, Go, Brazil. The diagnosis of allergic rhinitis was based on the international guidelines [1, 3] and that of asthma followed the GINA executive summary [12]. As inclusion criteria, children should have (i) clinical history of respiratory symptoms related to the house dust exposure; (ii) positive skin prick test (SPT) to *D. pteronyssinus* allergen extract; (iii) presence of serum IgE to *D. pteronyssinus* allergens determined by ELISA. The exclusion criteria were children with previous specific allergen immunotherapy, cardiovascular or malignant diseases, the presence of upper airway infections in the last 30 days prior to the study, the use of antihistamines within the previous week, and the use of oral or topic corticosteroids within the previous 2 to 3 weeks. Therefore, allergic children that enrolled in the study were under no influence of these treatment conditions. As control group, 14 nonallergic healthy children, with no symptoms or clinical history of allergic diseases and negative SPT to a panel of standardized aeroallergens, were selected among age- and socioeconomic status-matched children attended in the Pediatric Service of the Clinic Hospital of the Federal University of Uberlândia, Brazil. The study was approved by the Ethics Committee on Human Research at the Federal University of Uberlandia and informed consent was obtained from the children's parents.

### 2.2. Mite Allergen Extract

*D. pteronyssinus* (Dpt) crude extract was obtained from mite bodies and feces as described elsewhere [13]. Briefly, mite powder was triturated in liquid nitrogen and allergens were extracted in 5 mM borate-buffered saline containing protease inhibitors. After centrifugation (20,000 ×g for 45 min at 4°C), the supernatant was dialyzed, filtered through a 0.22 *μ*m membrane and protein concentration was determined [14]. For SPT, Dpt crude extract was adjusted to a protein concentration of 2 mg/mL in phosphate-buffered saline (PBS, pH 7.0) containing 0.4% phenol plus 50% glycerol and stored at 4°C until use. 

### 2.3. Skin Prick Test

All children underwent SPT according to European Academy of Allergology and Clinical Immunology guidelines [15], with a panel of aeroallergens, as follows: mite (*D. pteronyssinus, D. farinae, and B. tropicalis*) extracts prepared as previously described [13] and standardized commercial extracts of cockroaches (*Blattella germanica* and *Periplaneta americana*), mold (*Alternaria alternata*) and pet danders (*Felis domesticus* and *Canis familiaris*) (FDA Allergenic Ltda, Rio de Janeiro, RJ, Brazil). A histamine solution at 10 mg/mL and the glycerol-buffered diluent of the allergen preparation were used as positive and negative controls, respectively, (IPI/ASAC, São Paulo, Brazil). Skin reactions were read after 15 min and a wheal mean diameter 3 mm larger than the negative control was considered a positive SPT result. 

### 2.4. Serum and Saliva Samples

Venous blood and saliva samples were obtained from all children in parallel to SPT. Serum samples were collected after centrifugation (700 ×*g *for 10 min) of blood samples (5 mL) and were then stored in aliquots at −20°C until serological assays. Unstimulated saliva samples (1–1.5 mL) were collected from the oral cavity of the children using standard Salivette (Sarstedt AG & Co., Numbrecht, Germany) devices during approximately 3 min as described elsewhere [16]. Samples were immediately placed on ice and centrifuged (3.000 ×*g* for 10 min a 4°C). Supernatants were collected and stored in aliquots at −20°C until assays. 

### 2.5. Serum and Salivary IgE, IgA, and IgG4 Levels to *D. pteronyssinus* Allergens

Serum and salivary IgE, IgA, and Ig4 levels to *D. pteronyssinus* allergens were determined by using conventional ELISA (for Dpt allergen) or reverse ELISA (for Der p1 and Der p2 allergens) as previously described [17]. 

For Dpt allergen, high-binding microtiter plates were coated overnight at 4°C with Dpt crude extract (20 *μ*g/mL) and then blocked with bovine serum albumin (BSA) at 1% (for IgE and IgA) or 0.1% (for IgG4) in PBS plus 0.05% Tween-20. Serum samples were diluted 1 : 2 (IgE), 1 : 50 (IgA), or 1 : 10 (IgG4) in the respective blocking buffers and incubated for 2 h at 37°C. Likewise, saliva samples were diluted 1 : 2 (IgE), 1 : 20 (IgA), or 1 : 10 (IgG4) in the respective blocking buffers and incubated for 2 h at 37°C. Subsequently, plates were incubated with biotinylated secondary antihuman IgE (1 : 1,000; Kirkegaard & Perry Laboratories, Gaithersburg, Md, USA), antihuman IgA (1 : 5,000 for serum and 1 : 8,000 for saliva samples; Sigma Chemical Co., St. Louis, Mo, USA) or antihuman IgG4 (1 : 5,000 for serum and 1 : 3,000 for saliva samples; Sigma Chemical Co.) antibodies for 1 h at 37°C, followed by streptavidin-peroxidase (1 : 500; Sigma Chemical Co.) for 30 min at 37°C. The assay was developed by adding 0.01 M 2,2′-azinobis (3-ethylbenzthiazoline-6-sulfonic acid) (ABTS) and 0.03% H_2_O_2_. Optical density (OD) was determined at 405 nm and antibody levels were expressed in ELISA index (EI) for IgE and IgG4 antibodies, as described earlier [18], according to the following formula: EI = OD of the test sample/cut off, where the cut off was established as the mean OD values of negative control serum or saliva samples plus 3 standard deviations. EI values >1.2 were considered positive for IgE and IgG4 antibodies. For IgA detection, antibody levels were expressed as arbitrary units per milliliter (AU/mL) and calculated on the basis of a reference curve constructed with a pool of serum or saliva samples with high levels of IgA antibodies to Dpt and established as containing 1000 AU/mL at double dilutions ranging from 1000 to 0.977 AU/mL by using the Microplate Manager 4.0 (BioRad Laboratories Inc., Hercules, USA) software. 

For Der p1 and Der p2 allergens, high-binding microtiter plates were coated with mouse monoclonal antibody to Der p1 (clone 5H8; Indoor Biotechnologies, Charlottesville, Va, USA) or to Der p2 (clone 1D8; Indoor Biotechnologies, Charlottesville, Va, USA) at 1.0 *μ*g/well and then incubated subsequently with Dpt extract, serum samples, biotinylated secondary antihuman IgE (1 : 1,000), antihuman IgA (1 : 3,000) ou antihuman IgG4 (1 : 3,000), streptavidin-peroxidase conjugate, and enzyme substrate as described above for conventional ELISA. Levels of IgE and IgG4 to major allergens (Der p1 and Der p2) were expressed as ELISA index (EI) as established for conventional ELISA. To obtain the reference curve of IgA anti-Der p1 and anti-Der p2, it was used in each ELISA plate a pool of serum or saliva samples with high levels of specific IgA antibodies for each allergen that were arbitrarily established as containing 1000 AU/mL. 

It is important to make clear that sera from nonallergic subjects were randomly included in the same ELISA plate along with sera from allergic patients. In addition, two positive quality-control sera and three negative control sera were included in each plate in order to calculate the cut-off value of the reaction by ELISA index (EI) mite-specific IgE and IgG4 antibodies. 

### 2.6. Statistical Analysis

Statistical analysis was performed using the GraphPad Prism 5.0 software (GraphPad Software, Inc., San Diego, Calif, USA). As the antibody levels were not normally distributed, comparisons were analyzed using nonparametric tests. Differences between groups were analyzed using the Mann-Whitney test. Differences in the percentages of positive samples for IgG4 antibodies to mite allergens between groups were analyzed by the Fisher exact test. Correlation between antibody levels was analyzed by the Spearman correlation test. Differences were considered to be statistically significant when *P* < 0.05.

## 3. Results

### 3.1. Subject Data

The demographic and clinical characteristics of the study subjects are shown in Table 1. From a total of 86 children analyzed, 72 had allergic rhinitis (M/F: 42/30; mean ± SD age: 10.0 ± 3.1 yr) and 14 were nonallergic healthy children (M/F: 9/5; mean ± SD age: 10.6 ± 2.6 yr). There was no significant difference regarding the sex and age between the allergic and nonallergic children (*P* > 0.05). The frequency of allergic children with rhinitis alone (71%) was higher than rhinitis associated with asthma (29%) (*P* < 0.0001). SPT results to aeroallergen extracts revealed higher concomitant sensitization to the mites *D. pteronyssinus* and *D. farinae* (96%) than to *Blomia tropicalis* (74%) and to other aeroallergens (<55%) (*P* < 0.0001), and only the sensitization to *Alternaria alternata* (14%) was not significantly different between the allergic and nonallergic groups (*P* = 0.3559). 

### 3.2. IgE Levels to Dpt, Der p1 and Der p2 Allergens

**Figure 1:**
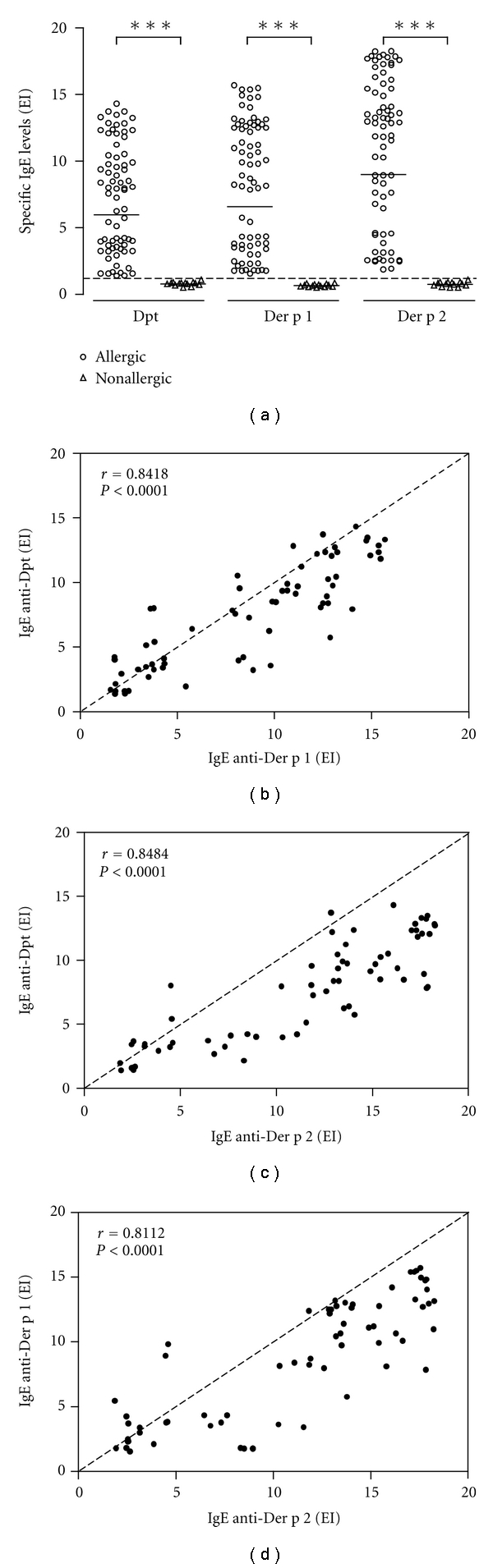
(a) Levels of IgE antibodies to *Dermatophagoides pteronyssinus* (Dpt) crude extract and its major allergens (Der p1 and Der p2) in serum samples from allergic (*n* = 72) and nonallergic (*n* = 14) infants. Data are expressed in ELISA index (EI) and the geometric mean for each allergen is indicated by horizontal bars. The dashed line indicates the cutoff of the reaction (EI > 1.2). ***P < 0.0001 as determined by the Mann-Whitney *U* test. (b), (c), and (d) Correlation between levels of serum IgE anti-Dpt versus anti-Der p1 (b), IgE anti-Dpt versus anti-Der p2 (c), and IgE anti-Der p1 versus anti-Der p2 (d). The Spearman correlation coefficient (*r*) and statistical significance are also indicated.

Levels of serum IgE antibodies to Dpt extract were significantly higher in allergic (EI geometric mean [gm]: 5.97) than nonallergic (EI gm: 0.79; *P* = 0.0004) children. Similar results were observed for levels of IgE to Der p1 (EI gm: 6.59 versus 0.67; *P* < 0.0001) and Der p2 (EI gm: 9.00 versus 0.75; *P* < 0.0001) (Figure 1(a)). Nonetheless, levels of specific salivary IgE were undetectable in both allergic and nonallergic children (data not shown). 

When analyzing correlation between IgE levels to Dpt and its major allergens in allergic children, levels of IgE anti-Dpt showed strong positive correlation with IgE anti-Der p1 (*r* = 0.8418; *P* < 0.0001) (Figure 1(b)) and IgE anti-Der p2 (*r* = 0.8484; *P* < 0.001) (Figure 1(c)). Similarly, IgE levels anti-Der p1 positively correlated with IgE anti-Der p2 (*r* = 0.8112; *P* < 0.0001) (Figure 1(d)).

### 3.3. IgA Levels to Dpt, Der p1 and Der p2 Allergens

**Figure 2:**
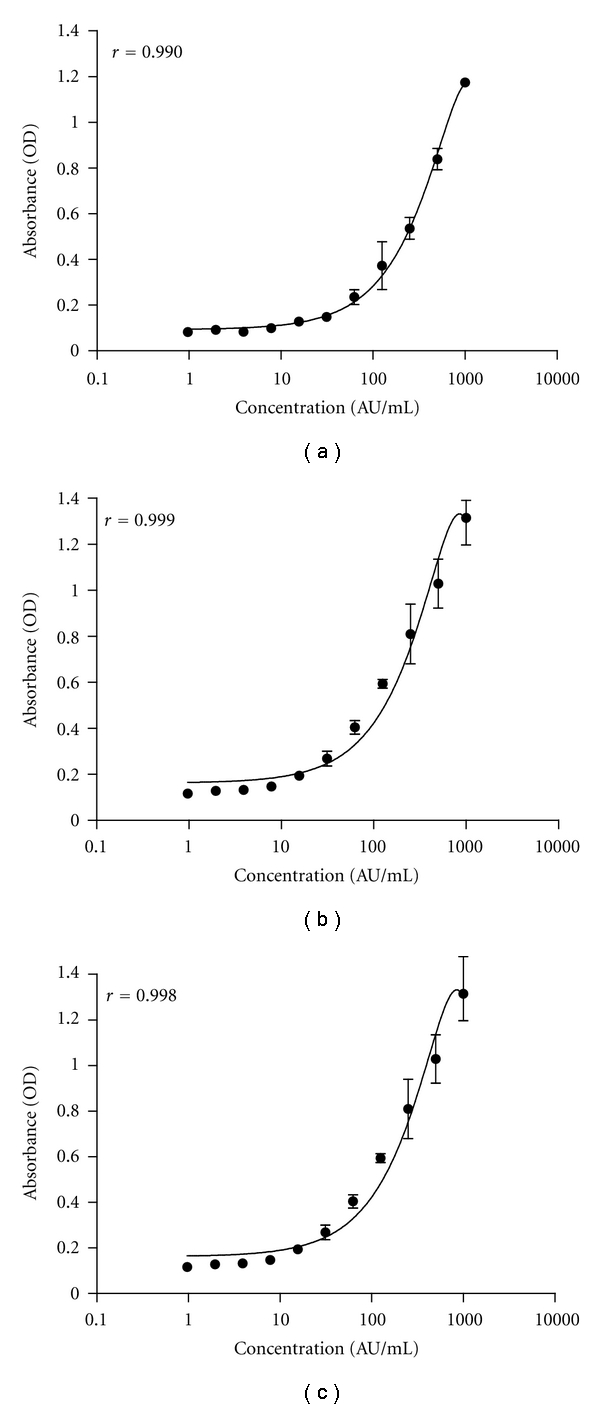
Representative reference curves of IgA antibodies to *Dermatophagoides pteronyssinus* (Dpt) (a) and its major allergens Der p1 (b) and Der p2 (c) in saliva samples. A pool of saliva samples with high levels of specific IgA antibodies for each allergen and established as containing 1000 arbitrary units per milliliter (AU/mL) was used for each reference curve, using serial double dilutions ranging from 1000 to 0.977 AU/mL. The curves were constructed with the GraphPad Prism 5.0 software by plotting the absorbance (OD) and concentration (AU/mL) values, using the quadratic curve fit. The correlation coefficients (*r*) are indicated and the detection limits for these assays were 7.8 AU/mL for IgA anti-Dpt, and 3.9 AU/mL for IgA anti-Der p1 and IgA anti-Der p2.

Representative reference curves of IgA antibodies to Dpt and its major allergens, Der p1 and Der p2, in saliva samples are shown in Figure 2. The correlation coefficients were above 0.990 for all the reference curves. The detection limits for these assays were 7.8 AU/mL for IgA anti-Dpt, and 3.9 AU/mL for IgA anti-Der p1 and IgA anti-Der p2 in both serum and saliva samples. The inter- and intra-assay reproducibility of these assays was evaluated by calculating the respective coefficients of variation that were below 15%.

**Figure 3:**
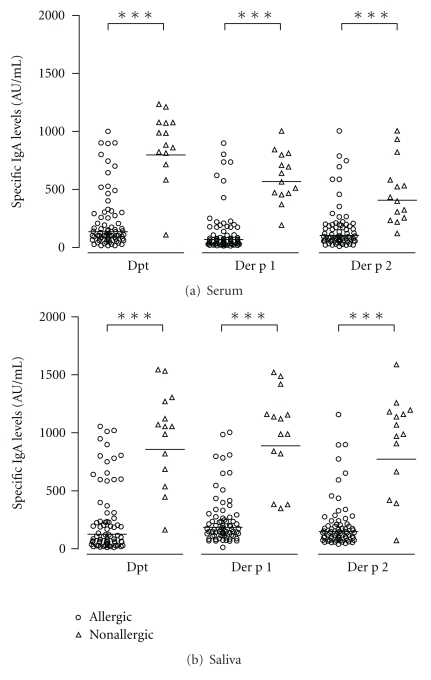
Levels of IgA antibodies to *Dermatophagoides pteronyssinus* (Dpt) crude extract and its major allergens (Der p1 and Der p2) in serum (a) and saliva (b) samples from allergic (*n* = 72) and nonallergic (*n* = 14) infants. Data are expressed in arbitrary units per milliliter (AU/mL) and the geometric mean for each allergen is indicated by horizontal bars. ***P < 0.0001 as determined by the Mann-Whitney *U* test.

Levels of serum IgA antibodies to Dpt, Der p1 and Der p2, allergens were significantly higher in nonallergic (gm: 791 AU/mL, 568 AU/mL, and 407 AU/mL, resp.) than allergic (gm: 135 AU/mL, 69 AU/mL, and 103 AU/mL, resp.; *P* < 0.0001) children (Figure 3(a)). Similar results were observed for salivary IgA levels to Dpt, Der p1 and Der p2, in nonallergic (gm: 858 AU/mL, 889 AU/mL, and 774 AU/mL, resp.) as compared to allergic (gm: 126 AU/mL, 186 AU/mL, and 150 AU/mL, resp.; *P* < 0.0001) children (Figure 3(b)). 

**Figure 4:**
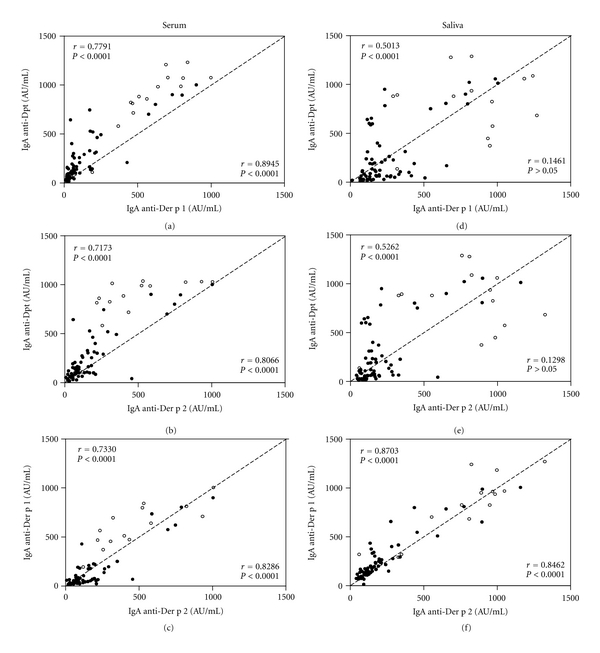
Correlation between levels of IgA antibodies to *Dermatophagoides pteronyssinus* (Dpt) crude extract and its major allergens (Der p1 and Der p2) in serum (a–c) and saliva (d–f) samples from 72 allergic (•) and 14 nonallergic (∘) children. Data are expressed in arbitrary units per milliliter (AU/mL) and the dashed line indicates a perfect correlation. The Spearman correlation coefficients (*r*) and statistical significance are also indicated for allergic (on the upper left corner) and nonallergic (on the lower right corner) children.

Correlation between IgA levels to Dpt and its major allergens, Der p1 and Der p2, in serum or saliva samples from allergic and nonallergic children, are shown in Figure 4. There was a strong positive correlation between levels of serum IgA to Dpt and its major allergens as well as between Der p1 and Der p2 allergens in both groups, particularly in nonallergic children (Figures 4(a), 4(b), and 4(c)). On the other hand, salivary IgA levels to Dpt showed a moderate positive correlation with IgA anti-Der p1 or anti-Der p2 in the allergic group, whereas these parameters were not correlated in the nonallergic group (Figures 4(d) and 4(e)). In contrast, levels of salivary IgA anti-Der p1 and anti-Der p2 exhibited a strong positive correlation in both allergic and nonallergic groups (Figure 4(f)). 

No correlation was found between serum and salivary IgA levels to Dpt, Der p1 and Der p2 allergens, in both allergic and nonallergic children (Table 2). 

### 3.4. IgG4 Levels to Dpt, Der p1 and Der p2 Allergens

**Figure 5:**
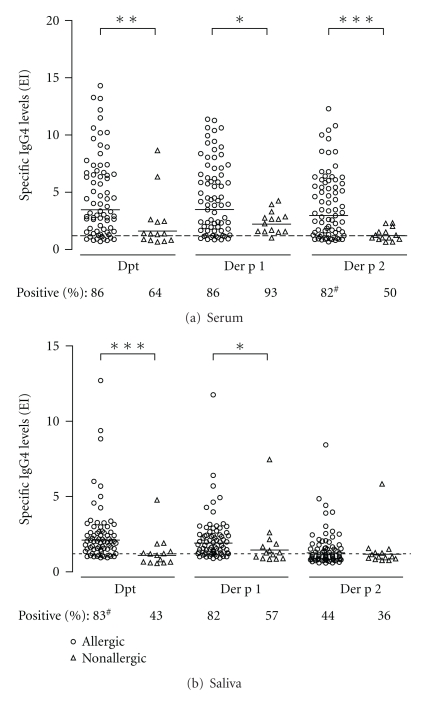
Levels of IgG4 antibodies to *Dermatophagoides pteronyssinus *(Dpt) crude extract and its major allergens (Der p1 and Der p2) in serum (a) and saliva (b) samples from allergic (*n* = 72) and nonallergic (*n* = 14) infants. Data are expressed in ELISA index (EI) and the geometric mean for each allergen is indicated by horizontal bars. The dashed line indicates the cut off of the reaction (EI > 1.2) and percentages of positive IgG4 samples for each allergen are also indicated. *P < 0.05; **P < 0.001; ***P < 0.0001 as determined by the Mann-Whitney *U* test; ^#^Statistical significance determined by the Fisher exact test (*P* < 0.05).

Levels of serum IgG4 antibodies to Dpt, Der p1 and Der p2 allergens, were significantly higher in allergic (EI gm: 3.47, 3.49, and 2.97, resp.) than nonallergic (EI gm: 1.62, 1.62, and 1.22, resp.; *P* < 0.05) children (Figure 5(a)). Similar results were observed for salivary IgG4 levels to Dpt and Der p1 in allergic (EI gm: 2.15 and 1.93, resp.) as compared to nonallergic (EI gm: 1.09 and 1.45, resp.; *P* < 0.05) children (Figure 5(b)), but levels of salivary IgG4 to Der p2 did not show significant differences between allergic and nonallergic children (*P* > 0.05) (Figure 5(b)). Percentage of positive serum samples for IgG4 to Dpt and Der p1 were not significantly different between allergic and nonallergic groups (*P* > 0.05), but allergic children showed higher positive rate of serum IgG4 to Der p2 than nonallergic ones (82% versus 50%; *P* = 0.016) (Figure 5(a)). In saliva samples, it was observed higher positivity for IgG4 to Dpt and Der p1 in allergic (83% and 82%, resp.) than nonallergic children (43% and 57%, resp.), although significant differences were found only for Dpt (*P* = 0.002). Positivity of salivary IgG4 to Der p2 was not significantly different between the allergic and nonallergic groups (Figure 5(b)).

**Figure 6:**
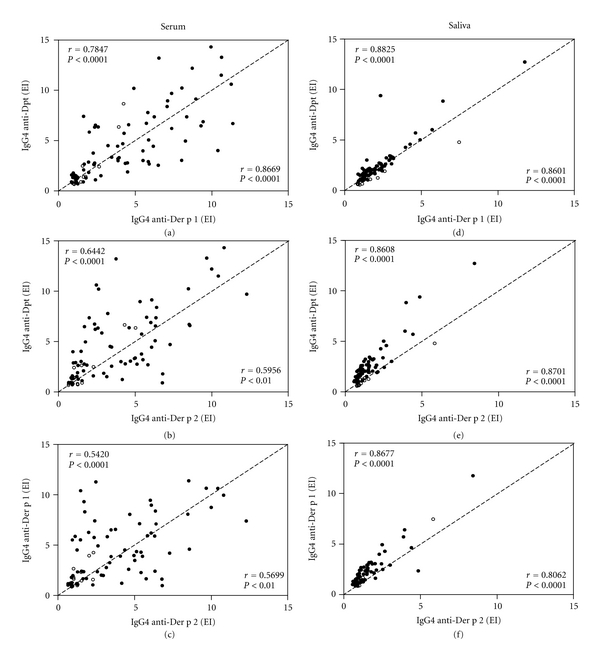
Correlation between levels of IgG4 antibodies to *Dermatophagoides pteronyssinus* (Dpt) crude extract and its major allergens (Der p1 and Der p2) in serum (a–c) and saliva (d–f**) **samples from 72 allergic (•) and 14 nonallergic (∘) infants. Data are expressed in ELISA index (EI) and the dashed line indicates a perfect correlation. The Spearman correlation coefficients (*r*) and statistical significance are also indicated for allergic (on the upper left corner) and nonallergic (on the lower right corner) subjects.

Correlation between IgG4 levels to Dpt and its major allergens, Der p1 and Der p2, in serum or saliva samples from allergic and nonallergic children are shown in Figure 6. There was a strong positive correlation between levels of serum IgG4 to Dpt and Der p1 in both groups, particularly in nonallergic children (Figure 6(a)), whereas a moderate positive correlation was observed for serum IgG4 to Dpt versus Der p2 as well as between Der p1 versus Der p2 in the two groups (Figures 6(b), and 6(c)). On the other hand, salivary IgG4 levels to Dpt, Der p1 and Der p2 were strongly correlated among themselves in both allergic and nonallergic groups (Figures 6(d), 6(e), and 6(f)). 

When analyzing the correlation between serum and salivary IgG4 levels to Dpt, Der p1 and Der p2 allergens, it was found a weak, even though significant positive correlation for all allergens only in the group of allergic children (*P* < 0.05) (Table 2). 

### 3.5. Comparison between Serum IgG4, IgA, and IgE levels to Dpt, Der p1 and Der p2 Allergens

Serum levels of IgG4, IgA, and IgE to Dpt, Der p1 and Der p2, were analyzed with regards to correlation between IgG4 versus IgE, IgA versus IgE, and IgA versus IgG4 levels to each allergen in allergic and nonallergic groups (Table 3). Regarding the correlation between specific IgG4 versus IgE levels, a significant positive correlation was found for antibodies to Dpt (*r* = 0.2351; *P* < 0.05) and Der p2 (*r* = 0.3585; *P* < 0.001) only in the allergic group. Similar correlation was observed between specific IgA versus IgE to Dpt (*r* = 0.3533; *P* < 0.001), Der p1 (*r* = 0.3614; *P* < 0.001), and Der p2 (*r* = 0.2327; *P* < 0.05) allergens. Concerning the correlation between specific IgA versus IgG4 levels, a significant positive correlation was observed only to Dpt (*r* = 0.3122; *P* < 0.001) in the allergic group whereas a significant negative correlation was seen for Dpt (*r* = −0.5824; *P* < 0.05) and Der p1 (*r* = −0.6799; *P* < 0.001) in the nonallergic group. 

## 4. Discussion

Although children with allergic rhinitis sensitized to HDM are known to have increased levels of allergen-specific serum IgE, the presence of different antibody isotypes in saliva, such as IgE, IgA, and IgG subclasses as well as the relationship between serum and salivary antibodies or among antibody isotypes are not yet well established. In addition, with the increasing advance in approaches of sublingual immunotherapy for allergic rhinitis, the measurement of HDM-specific IgE, IgA, or IgG4 antibodies in saliva samples might be a potential tool for monitoring the immunological changes during the treatment. 

In the present study, children with allergic rhinitis had high serum IgE levels to Dpt and its major allergens, Der p1 and Der p2, which were strongly correlated among themselves. In addition, it was observed a tendency of increased IgE levels to Der p2, demonstrating that children were more sensitized to Der p2 than Der p1. Accordingly, previous studies showed that IgE levels against Der p2 were always higher than those against Der p1 in patient sera, confirming that Der p2 is a more immunologically active molecule than Der p1 in inducing IgE synthesis [19, 20]. On the other hand, specific salivary IgE levels were undetectable in both allergic and nonallergic children, confirming previous report that IgE antibodies were not found in saliva [21]. These findings suggest that mite-specific IgE antibodies were not produced locally at the oral mucosa nor passively transferred from serum or nasal secretions, even though it is well known that specific IgE is an important class of immunoglobulin in the nasal mucosa and appears to be locally produced [21, 22]. Alternatively, negative salivary IgE data could be due to low recovery of specific IgE in saliva samples collected by the Salivette technique if mite-specific IgE antibodies were present in very low concentrations in saliva, although other antibody isotypes such as salivary specific IgA and IgG4 were efficiently detected using the same procedure. Accordingly, standard Salivette devices have been used for a long time for collection of saliva samples that can be stored for several days in the own Salivette or dried onto filter paper with good IgA antibody recovery [16]. However, further studies could be conducted for the validation of total IgA or IgE recovery as well as the day-to-day variation using the Salivette device for saliva sample collection. 

In the current study, IgA levels to Dpt and its major allergens, Der p1 and Der p2, were higher in both serum and saliva samples from nonallergic than allergic children, suggesting a protective role of allergen-specific IgA antibodies for the development of respiratory allergic diseases. It has been proposed that low levels of salivary IgA are associated with the development of allergy [9]. Decreased total IgA has already been described in the saliva of children with atopy [23] as well as defective salivary IgA responses against the respiratory syncytial virus were found in symptomatic allergic infants [24]. In another previous study, nonatopic healthy individuals had increased Der p1-specific IgA production in serum, but not for specific IgE antibodies [4]. The increase in specific IgA in the serum coincided with increased TGF-*β*, accounting for the role of IgA and TGF-*β* in peripheral mucosal immune responses to allergens in healthy individuals [25]. In addition, the presence of specific serum and salivary IgA in patients with allergic rhinitis appears to have a concurrent immunoregulatory mechanism by activating TGF-producing T regulatory cells in the attempt to normalize the allergic reactions [25].

Contrasting to our findings, Kitani et al. [26] demonstrated that mite-specific IgA antibodies in serum and sputum samples were significantly higher in mite-sensitive patients than in normal controls and mite-insensitive asthmatic patients. Also, infants who developed allergy up to 2 years of age tended to have higher levels of total IgA and cat allergen-specific IgA in saliva than nonallergic children [9]. Another early study was also unable to detect IgA or IgE antibodies to *D. pteronyssinus* in sera from nonallergic persons, even though using a partially purified fraction of mite extract in antigen-binding assays [27]. A more recent study comparing Der p1-specific antibody levels in children with allergic airway disease and healthy controls showed that specific IgA levels in allergic children were not significantly different from controls, but when the IgA/IgE ratio was investigated, it was found to be significantly lower in children with asthma, but not in children with allergic rhinitis as compared to controls [4]. 

Analyzing the correlation between levels of specific IgA to Dpt and its major allergens, there was a strong correlation of serum IgA levels among the three allergens, in both allergic and nonallergic groups. In contrast, salivary IgA levels to Dpt showed a moderate correlation with Der p1 and Der p2 only in allergic children. In addition, no correlation was found between serum and salivary levels of IgA to the three allergens in both groups. These findings suggest that systemic immune responses are much more stimulated than secretory immune responses in both allergic and nonallergic children, and that IgA seems to be locally produced rather than passively transferred from the serum. Accordingly, it is long-time known that the great bulk of IgA antibodies in secretions is locally produced [21, 28], although primed IgA-producing B cells can migrate to distant mucosal surfaces and, as result, the site of production of IgA antibodies may not indicate the site of response to antigen [28]. It is interesting to reinforce that the presence of high levels of specific IgA, particularly in the serum of nonallergic children, may be responsible to prevent allergen sensitization. In the allergic group, however, significant correlations between levels of IgA versus IgE to the three allergens were observed, supporting that specific IgA can be induced for inhibiting the binding of allergens in IgE-producing plasma cells as already previously described [9]. It has been also reported that IgA deficiency was associated with the development of atopy within the first year in the offspring of reaginic patients, reinforcing the importance of the IgA antibody synthesis for preventing the development of atopic diseases [29]. 

In addition to IgA antibody, previous studies have demonstrated a protective activity for IgG subclasses, particularly IgG4, which is produced as result of a long-time natural allergen exposure or a specific allergen immunotherapy [13, 30]. In the present study, serum IgG4 levels to Dpt and its major allergens were higher in allergic than nonallergic, reinforcing that antigens that induce IgE antibodies are also good inducers of IgG4 antibodies [31]. These findings were also supported, in the present study, by the positive correlation found between serum IgG4 versus IgE levels to Dpt and mostly to Der p2 in allergic, but not in nonallergic children. Our results agree with a number of studies reporting good correlations between specific IgE and IgG4 levels after natural exposure to allergens in patients with allergic airway diseases [13, 32], although there is a report in Japan that did not find such correlation between levels of IgG4 and IgE to Der p2 allergen [33]. Interestingly, our results showed negative correlations between serum IgG4 versus IgA levels to Dpt and Der p1 only in nonallergic children, reinforcing that the presence of specific IgA appears to have a key role for the healthy immune response to mucosal allergens. 

Likewise, salivary IgG4 levels to Dpt and Der p1, but not to Der p2, were higher in allergic than nonallergic children, supporting our previous report that Der p1 is a more immunogenic molecule than Der p2 in inducing IgG4 synthesis [20]. In addition, the moderate positive correlation between serum and salivary IgG4 levels to all allergens found in allergic children, conversely to that observed for specific IgA correlation, reflects that IgG4 is likely passively transferred rather than locally produced. 

The association of IgG4 with protective activity is related to its function as blocking antibody or marker of tolerance induction. The mechanisms for blocking antibody are the competition for allergen between IgG4 and cell-bound IgE antibodies [31] and IgE-facilitated allergen presentation, in which this IgE-facilitation is prevented in the presence of IgG4 antibody because of competition between IgE and IgG4 antibodies, resulting in a decreased sensitivity of T cells and consequently in a suppression of the late-phase reactions [34, 35]. As marker of tolerance induction, IgG4 antibody measurements may be particularly valuable in follow-up studies, where a considerable increase in IgG4 levels can be a strong indicator of the activation of tolerance-inducing mechanisms [31]. This protective activity may be a significant underlying mechanism for the efficacy of specific-allergen immunotherapy.

Taken together, we can conclude that mite-specific IgA antibodies predominate in the serum and saliva of nonallergic children whereas mite-specific IgE and IgG4 antibodies are prevalent in allergic children. Also, levels of serum IgE to total Dpt extract and its major components are positively correlated with serum specific IgG4 or IgA levels in allergic children, but serum IgA and IgG4 levels to Dpt and Der p1 are negatively correlated in nonallergic children. Therefore, the presence of specific IgA appears to have a key role for the healthy immune response to mucosal allergens. Also, specific IgA measurements in serum and/or saliva may be useful for monitoring activation of tolerance-inducing mechanisms during allergen-specific immunotherapeutic procedures, especially sublingual immunotherapy.

## Figures and Tables

**Table 1:** Demographic and clinical characteristics of the study subjects.

Characteristics	Groups		
	Allergic	Nonallergic	**P** value
Number of subjects	72	14	—
Age (yr)	10.0 ± 3.1	10.6 ± 2.6	N.S.^a^
Mean ± SD			
Sex (M : F)	42 : 30	9 : 5	N.S.^a^
Clinical diagnosis (*n*, %)			
Rhinitis	51 (71%)	0	<0.0001^b^
Rhinitis and asthma	21 (29%)	0	<0.0001^b^
Positive skin prick test (*n*, %)^§^			
*Dermatophagoides pteronyssinus*	72 (100%)	0	<0.0001^b^
*Dermatophagoides farinae*	69 (96%)	0	<0.0001^b^
*Blomia tropicalis *	53 (74%)	0	<0.0001^b^
*Blattella germanica *	31 (43%)	0	0.0015^b^
*Periplaneta americana*	22 (31%)	0	0.0168^b^
*Alternaria alternata*	10 (14%)	0	N.S.^b^
*Felis domesticus*	27 (37%)	0	0.0039^b^
*Canis familiaris*	37 (52%)	0	0.0002^b^

^§^Skin prick test to aeroallergen extracts was considered positive for a mean diameter of wheal size larger than 3 mm than negative control (saline diluent); N.S: nonsignificant.

^a^Determined by the Student *t*-test.

^b^Determined by the Fisher exact test.

**Table 2:** Correlation between serum and salivary levels of IgA and IgG4 to *Dermatophagoides pteronyssinus* (Dpt) crude extract and its major allergens (Der p1 and Der p2) in allergic and nonallergic children.

Groups	Serum versus saliva					
	IgA	IgG4	IgA	IgG4	IgA	IgG4
	anti-Dpt	anti-Dpt	anti-Der p1	anti-Der p1	anti-Der p2	anti-Der p2
Allergic	*r* = 0.1802	*r* = 0.3667	*r* = −0.0519	*r* = 0.3551	*r* = −0.0547	*r* = 0.2901
(*n* = 72)	*P* > 0.05	*P < 0.001	*P* > 0.05	*P < 0.001	*P* > 0.05	*P < 0.05

Nonallergic	*r* = 0.2640	*r* = −0.0396	*r* = 0.3407	*r* = 0.1894	*r* = 0.5209	*r* = 0.2970
(*n* = 14)	*P* > 0.05	*P* > 0.05	*P* > 0.05	*P* > 0.05	*P* > 0.05	*P* > 0.05

*r* = Spearman correlation coefficient; *statistical significance was set for *P* < 0.05.

**Table 3:** Correlation between serum IgG4, IgA, and IgE levels to *Dermatophagoides pteronyssinus* (Dpt) crude extract and its major allergens (Der p1 e Der p2) in allergic and nonallergic children.

Correlation	Groups	
	Allergic	Nonallergic
IgG4 versus IgE for Dpt	0.2351*	0.3484
IgG4 versus IgE for Der p1	0.1921	0.2310
IgG4 versus IgE for Der p2	0.3585**	0.4956
IgA versus IgE for Dpt	0.3533**	−0.3815
IgA versus IgE for Der p1	0.3614**	0.0285
IgA versus IgE for Der p2	0.2327*	−0.0418
IgA versus IgG4 for Dpt	0.3122**	−0.5824*
IgA versus IgG4 for Der p1	0.1172	−0.6799**
IgA versus IgG4 for Der p2	0.1858	0.0813

Data are expressed as coefficient of correlation of Spearman (*r*) with statistical significance (*P < 0.05; **P < 0.001).
